# Simulation of Jetting in Injection Molding Using a Finite Volume Method

**DOI:** 10.3390/polym8050172

**Published:** 2016-05-04

**Authors:** Shaozhen Hua, Shixun Zhang, Wei Cao, Yaming Wang, Chunguang Shao, Chuntai Liu, Binbin Dong, Changyu Shen

**Affiliations:** National Engineering Research Center of Mold & Die, Zhengzhou University, Zhengzhou 450002, China; hsz1024@163.com (S.H.); zsxzzu@zzu.edu.cn (S.Z.); wangyaming@zzu.edu.cn (Y.W.); shaochg@zzu.edu.cn (C.S.); ctliu@zzu.edu.cn (C.L.); dongbinbin@zzu.edu.cn (B.D.); shency@zzu.edu.cn (C.S.)

**Keywords:** jetting, finite volume method, Cross-WLF, VOF Method, injection molding

## Abstract

In order to predict the jetting and the subsequent buckling flow more accurately, a three dimensional melt flow model was established on a viscous, incompressible, and non-isothermal fluid, and a control volume-based finite volume method was employed to discretize the governing equations. A two-fold iterative method was proposed to decouple the dependence among pressure, velocity, and temperature so as to reduce the computation and improve the numerical stability. Based on the proposed theoretical model and numerical method, a program code was developed to simulate melt front progress and flow fields. The numerical simulations for different injection speeds, melt temperatures, and gate locations were carried out to explore the jetting mechanism. The results indicate the filling pattern depends on the competition between inertial and viscous forces. When inertial force exceeds the viscous force jetting occurs, then it changes to a buckling flow as the viscous force competes over the inertial force. Once the melt contacts with the mold wall, the melt filling switches to conventional sequential filling mode. Numerical results also indicate jetting length increases with injection speed but changes little with melt temperature. The reasonable agreements between simulated and experimental jetting length and buckling frequency imply the proposed method is valid for jetting simulation.

## 1 Introduction

Jetting is an abnormal melt flow in injection molding, which impairs both the appearance and mechanical properties of molding products. Engineers try to eliminate this undesirable phenomenon by using the fan, lap, overlap gates, or adjusting process conditions, such as reducing injection speed or mold temperature. However, these methods are not always valid for complicated parts molding due to the lack of sound theory. It is necessary to explore the jetting mechanism so as to predict the critical values which probably triggers jetting.

People always expect the melt filling in sequential mode, *i.e.*, the melt front advances from near to far places according the distance away from the gate. However, when jetting happens, a stream of melt will firstly spout into the empty cavity, then the succeeding melt fills its surrounding empty space according to the sequential filling mode. This leads to physical differences at the interface between the jetting fluid and subsequent filling melt. The non-uniform melt flow also leads to the significant discrepancy in appearance and poor quality [1,2]. This phenomenon was first reported by White and Dee with a visualization method [3]. Then, Oda *et al.* [4] applied this method to explore the jetting law of isothermal and non-isothermal melt filling for conventional materials. They found jetting occurs only at a particular injection rate range and a geometrical scope, *i.e.*, the product of die swell ratio and mold gate depth should be less than the mold cavity depth. The injection speed scope which can induce jetting was found by Xie *et al.* [5]. Moreover, they also found jetting can be eliminated by adjusting the gate locations. For glass-fiber reinforced polyamide injection molding, jetting depends on fiber length, content, and injection speed as Oda reported [4]. This was validated by Akay and Barkley [6], who found jetting was related to the die swell ratio, shear rate, molecular orientation, and injection speed, and can be avoided by increasing the injection speed for glass-fiber reinforced polymer.

However, visualizing experiments can hardly reveal the jetting mechanism during injection molding due to lack of detailed physical data support. Therefore, people have to construct the theoretical and numerical methods to explore the intrinsic characteristics associated with flow fields. Cruickshank [7,8] theoretically and experimentally determined the two critical parameters that induced jet buckling. A three-dimensional marker-particle method was introduced by Tomé [9] to simulate three-dimensional buckling. Ville [10] studied the buckling oscillation and entrapped air phenomenon, and developed a code using the Level Set Method to simulate the direction of jet buckling and the location of the entrapped bubbles. Slim *et al.* [11] employed Stokes equations and the viscous plate model to analyze the buckling stability of a thin jet. Their results show the viscous plate model can describe the most unstable mode above onset. Oishi *et al.* [12] presented numerical simulations of the jet buckling problem using the eXtended Pom-Pom model, and found jet buckling was significantly influenced by the rheological properties of the fluid.

Almost all previous studies focused on the jetting-induced boundaries of injection speed, mold temperature, and other process conditions. The mechanism which induces jetting phenomenon can be seldom founded in the published literatures. This study aims to find the critical values of physical variables such as inertial, viscous forces which trigger jetting, and explore the influence of mold structures and process conditions. Therefore, the melt flow model was established first in terms of incompressible, viscous, non-isothermal flow. Then, variations of flow fields such as velocity, pressure, and temperature during filling were simulated with the finite volume method. Finally, comparisons between inertial force and viscous force were conducted to find the critical values which induce the jetting. The experiment was carried out to validate the proposed method.

## 2. Melt Flow Theory

### 2.1. Governing Equations

The polymer melt can be regarded as incompressible, viscous, and non-isothermal fluid during filling [13], so the governing equations can be written as:

(1) ∇⋅u=0

(2) $$\frac{\partial \left(\rho u\right)}{\partial t}+\nabla \cdot \left(\rho uu\right)=-\nabla p+\nabla \cdot \left(\eta \dot{\gamma }\right)+\rho g$$

(3) $$C_{p}\rho \left(\frac{\partial T}{\partial t}+u\cdot \nabla T\right)=\nabla \cdot \left(k\nabla T\right)+\eta {\dot{\gamma }}^{2}$$

where, ρ is the fluid density, u, *p*, *T* represent velocity, pressure, and temperature, and $C_{p}$, *k*, η, $\dot{\gamma }$ denote the melt specific heat, thermal conductivity, viscosity, and shear rate, respectively.

### 2.2. Boundary Conditions

Polymer melt flow at the entrance is specified as at a constant velocity, and a no-slip boundary is applied on the mold wall:

(4) $$u=u_{0}\text{ at the entrance}$$

(5) u=0 on the mold wall

The third boundary condition is applied to describe heat exchange between mold wall and melt:

(6) $$-k\frac{\partial T}{\partial n}=h_{a}\left(T-T_{w}\right)$$

where, *h*_a_ and *T*_w_ represent the heat exchange coefficient and mold wall temperature, respectively.

### 2.3. Trace of Melt Front

The melt front is tracked by the Volume Of Fluid (VOF) method. Define ϕ (0≤ϕ≤1) as the filling factor which represents the filling degree of a control volume. According to the mass conservation ϕ should satisfy the transport equation:

(7) $$\frac{\partial \phi }{\partial t}+\nabla \cdot \left(u\phi \right)=0$$

If the mold is well designed, the cavity air will flee smoothly without any accumulation. Thus, the pressure at the melt front is usually assumed to equal to atmospheric pressure. For computation convenience it is set to be zero:

(8) p=0

### 2.4. Viscous Model

The modified Cross model is employed to describe the viscosity varying with temperature, pressure, and shear rate:

(9) η=η01+(η0|γ˙|τ*)1−n

where *n* is the power-law index, and $\dot{\gamma }$ is the shear rate. Zero-shear-rate viscosity $\eta_{0}$ is calculated by the WLF equation:

(10) $$\eta_{0}\left(T,p\right)=D_{1}\exp\left\{-\frac{A_{1}\left(T-T^{*}\right)}{A_{2}+\left(T-T^{*}\right)}\right\}$$

where $T^{*}=D_{2}+D_{3}p$, $A_{2}={\tilde{A}}_{2}+D_{3}p$, and *n*, $\tau^{*}$, $A_{1}$, $D_{1}$, $D_{2}$, $D_{3}$ are material constants.

## 3. Numerical Method

Since the finite volume method can better maintain the conservation of the original differential equation compared with other numerical approaches, it was employed here to discretize the governing equations.

### 3.1. Discrete Momentum Equation

Integrating momentum equation partially by using of Gauss’ divergence theorem for diffusive and convective terms yields:

(11) $$\frac{\partial }{\partial t}\underset{V}{∭}\rho udV+\iint_{\partial V}\rho u_{n}udS-\iint_{\partial V}\eta \frac{\partial u}{\partial n}dS=\underset{V}{∭}\left(-\nabla p+\rho g\right)dV$$

In the finite volume method, P denotes a control volume with n faces, $F_{f}$ is volumetric flow rate in surface f, and $A_{f}$ denotes the area vector which points outward from the surface. Discretizing Equation (11) on the given control volume P gives:

(12) $${\left(\frac{\partial \left(\rho u\right)}{\partial t}\right)}_{P}V_{P}+\sum_{f=1}^{n}\rho_{f}F_{f}u_{f}-\sum_{f=1}^{n}\eta_{f}A_{f}\cdot {\left(\nabla u\right)}_{f}=\left(g\rho_{P}-{\left(\nabla p\right)}_{P}\right)V_{P}$$

here *V*_p_ is the volume of *P*, the symbol with subscript *f* represents the corresponding value convected through face *f*.

The one order implicit finite difference scheme and backward difference format are employed to discretize the transient term and the velocity gradient in Equation (12), respectively. After discretizing the equation on every control volume P and collecting the same terms, a set of algebraic equations can be written as:

(13) $$a_{P}u_{P}^{t+\Delta t}+\sum_{N\neq P}a_{N}u_{N}^{t+\Delta t}=S_{P}^{t}-{\left(\nabla p\right)}_{P}$$

where $S_{P}^{t}=g\rho_{P}+\frac{{\left(\rho u\right)}_{P}^{t}}{\Delta t}$, $a_{P}$ and $a_{N}$ are the coefficients for unknown velocity at control volume P and its neighbor, control volume *N*.

Then velocity can be determined in the following scheme:

(14) $$u_{P}^{t+\Delta t}=\frac{H(u^{t})}{a_{P}}-\frac{{\left(\nabla p\right)}_{P}}{a_{P}}$$

with $H(u^{t})=S_{P}^{t}-\sum a_{N}u_{N}^{t+\Delta t}$.

### 3.2. Discrete Continuity Equation

Integrating the continuity Equation (1) in the filled region *V*, then the volumetric integral can be transformed on the surround surface ∂V via Gauss’ formula:

(15) $$\iint_{\partial V}u_{n}dS=0$$

Using the two-dimensional finite volume method to discretize the boundary integral of Equation (15) gives the algebraic equation:

(16) $$\sum_{f}A_{f}\cdot u_{f}=0$$

Substitution of Equation (14) into Equation (16) yields:

(17) $$\sum_{f}A_{f}\cdot {\left[\frac{{\left(\nabla p\right)}_{P}}{a_{P}}\right]}_{f}=\sum_{f}A_{f}\cdot {\left(\frac{H(u)}{a_{P}}\right)}_{f}$$

Discretizing the pressure gradient with the finite difference method on every control volume P forms a set of algebraic equations about the coupled velocity u and pressure *p*. Since the coefficient $a_{P}$ depends on viscosity η and η is the function of u and *p*, then Equation (17) is nonlinear. The integral method for solving this kind of equations requires that a large-scale Jacobi matrix and its inverse matrix have to be calculated repeatedly [14]. For the sake of reducing the large amount of calculation and memory, an iterative scheme was proposed in this study to determine the unknown variables u and *p* separately. First, solve Equation (17) to determine the pressure *p* at the giving velocity u. Then calculate the pressure gradient ∇p on control volume P and substitute it into Equation (14) to get velocity solution u. Use the just-determined solution to update the coefficients in Equation (17) and solve it again. Repeat this process until both velocity and pressure are convergent. As the coefficient matrix about pressure in Equation (17) is symmetric, the stability and convergence of this iterative method is superior to the integral method whose coefficient matrix is asymmetric. Moreover, the iterative method reduces the memory because the velocity and pressure are determined separately.

### 3.3. Discrete Energy Equation

Integrating the energy Equation (3) in parts in the filled region *V* via Gauss’ divergence theorem gives:

(18) $$\underset{V}{∭}C_{p}\rho \frac{\partial T}{\partial t}dV+\iint_{\partial V}C_{p}\rho u_{n}TdS=k\iint_{\partial V}\frac{\partial T}{\partial n}dS+\underset{V}{∭}\eta {\dot{\gamma }}^{2}dV$$

Equation (18) was discretized with the similar method applied for Equation (11), except that the convective terms were dealt with upwind scheme [15]. Thus, the discreted equation can be written as:

(19) $$\frac{C_{p}\rho_{P}T_{P}^{t+\Delta t}-C_{p}\rho_{P}T_{P}^{t}}{\Delta t}+\frac{1}{2}\sum_{f}C_{p}\rho_{f}F_{f}^{t}T_{f}^{t+\Delta t}-\frac{1}{2}\sum_{f}k_{f}A_{f}^{t}\cdot {\left(\nabla T\right)}_{f}^{t+\Delta t}=\;{\left(S_{P}^{\prime }\right)}^{t}V_{P}-\frac{1}{2}\sum_{f}C_{p}\rho_{f}F_{f}^{t}T_{f}^{t}+\frac{1}{2}\sum_{f}k_{f}A_{f}^{t}\cdot {\left(\nabla T\right)}_{f}^{t}$$

where $F_{f}=A_{f}\cdot u_{f}$, $S_{P}^{\prime }=\eta {\dot{\gamma }}^{2}$. Equation (19) can be reduced to a simplified algebraic equation about temperature *T*:

(20) $$b_{P}T_{P}^{t+\Delta t}+\sum_{N\neq P}b_{N}T_{N}^{t+\Delta t}=S_{P}^{\prime \prime }$$

where, $S_{P}^{\prime \prime }={\left(S_{P}^{\prime }\right)}^{t}V_{P}-\frac{1}{2}\sum_{f}C_{p}\rho_{f}F_{f}^{t}T_{f}^{t}+\frac{1}{2}\sum_{f}k_{f}A_{f}^{t}\cdot {\left(\nabla T\right)}_{f}^{t}$, and $b_{P}$, $b_{N}$ are the coefficients corresponding to the control volume P and its neighbor control volume N, respectively.

As $b_{P}$ and $b_{N}$ depend on viscosity, and viscosity closely relates to temperature, the temperature and velocity are also coupled. The Successive Over-Relaxation (SOR) method is used to solve the coupled flow and thermal problems. First, solve the flow problem at the given temperatures to get the solutions of velocity and viscosity. Second, solve the thermal conducting problem to get the solution of temperature *T* using the just-determined flow field, and update the temperature with the SOR method, *i.e.*, $T^{t+△t}=\omega T^{t+△t}+\left(1-\omega \right)T^{t}$ (ω is relaxation factor). Finally, use these temperatures to solve the flow problem again. Repeat this iteration until both velocity and temperature are convergent.

This numerical scheme involves two-fold iterations. The inner iteration is carried out between velocity and pressure at given temperature. The outer iteration is performed between temperature and velocity. Our numerical simulations indicate the inner iteration number is much larger than that of the outer iteration because the coefficients in the momentum equations depend on the velocity itself but the coefficients in the energy equation do not depend on the temperature directly. Thus, the energy equation is usually regarded as a linear equation. The convergence difficulty of this kind of equation is much less than the nonlinear problem.

Similarly, the transport Equation (7) can be discretized with finite volume method as follows:

(21) $$\phi_{P}^{t+\Delta t}=\frac{\phi_{P}^{t}-\Delta t\sum_{f}F_{f}\phi_{f}^{t+\Delta t}}{V_{P}}$$

### 3.4. Time Step Determination

In order to ensure the stability and convergence of numerical algorithm, the Courant number is used to determine the time step:

(22) $$C=\left|\frac{u_{f}\cdot A_{f}}{d_{f}\cdot A_{f}}\right|\Delta t\leq 1$$

where $d_{f}$ is the vector between two element centers.

## 4. Results and Analysis

In this study, a rectangular plate of 120 mm × 60 mm × 4 mm is chosen as the molding product. The gate is located at one end of the product with dimensions of 6 mm × 6 mm × 2 mm. The polymer used in this study is polypropylene (PP, Polyfort FIPP MKF 4025, A. Schulman GMBH, Kerpen-Sindorf, Germanycity). The material constants are listed in Table 1. The gate points to the cavity directly, which can induce jetting easily. The simulations were performed by varying velocities and temperatures to investigate the jetting mechanism and the factors which influence the jetting evolution.

### 4.1. Jetting Evolution and the Induced Mechanism

**Figure 1:**
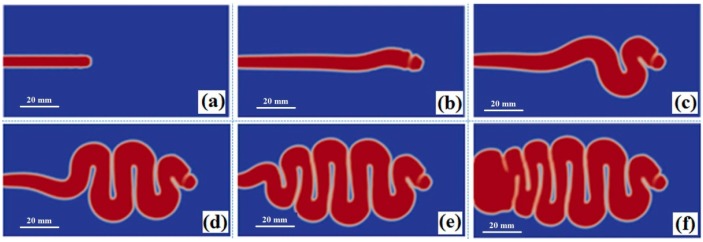
The simulated jetting evolution and buckling flows at (**a**) 0.2 s; (**b**) 0.4 s; (**c**) 0.6 s; (**d**) 1.0 s; (**e**) 1.3 s; and (**f**) 1.6 s.

In order to illustrate the jetting evolution the numerical simulations were first performed at an injection speed 200 mm/s, a melt temperature of 255 °C, and a mold temperature of 55 °C. Figure 1 shows the predicted jetting shapes in the cavity at different times. As the filling commences, the melt shoots straight into the cavity (see Figure 1a). Then the front starts bending, as Figure 1b exhibits. The succeeding melt continues to push the front melt, marching forward, and leads the melt to fold up and down. The melt filling exhibits the buckling flow (see Figure 1c–e). When the folding transfers to the gate, the melt filling switches to the conventional sequential filling mode, *i.e.*, filling from near to far places successively, see Figure 1f.

**Figure 2:**
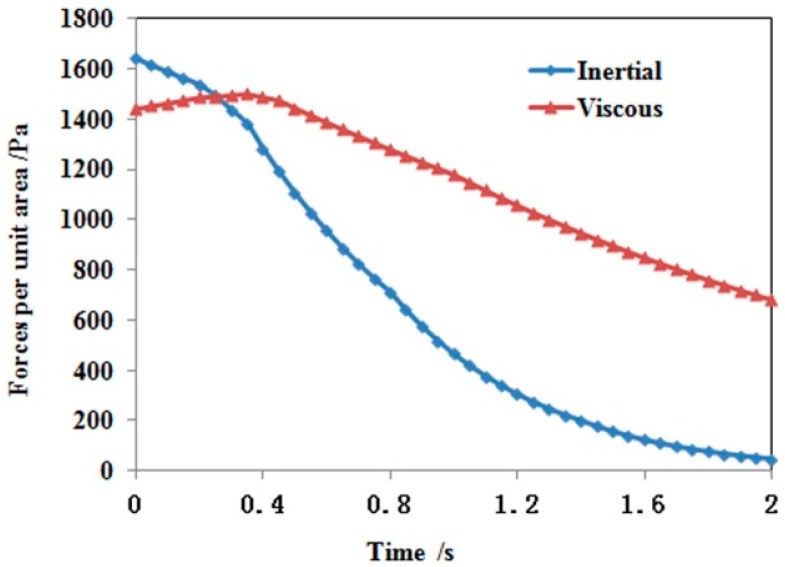
The comparison between inertial and viscous forces at the jetting front.

The melt flow in the cavity is closely related to viscous and inertial forces at the melt front. These two forces evolve in different manners during filling (see Figure 2). For convenience, the viscous stress is compared with the inertial force per unit area, calculated by $\rho \frac{Du}{Dt}\cdot V_{P}/S_{f}$ ($S_{f}$ is the total surround area of control volume $V_{P}$). The inertial force decreases gradually with time due to decreasing velocity. However the viscous stress first increases within the initial filling period, then decreases in the remaining filling time. Since the heat exchange between the melt and air leads the melt temperature at the interface to decrease 1.75 °C within 0.3 s of filling, the corresponding viscosity increases from 493.93 to 517.72 Pa∙s accordingly. Although the shear rate at the melt front changes little (about 2.91 1/s), the viscous force increases from 1143.87 to 1507.31 Pa which is larger than the inertial force (1435.75 Pa). Therefore, the inertial force is not large enough to drive the melt forward, and the front melt starts to fold under the rear melt. The melt filling exhibits a buckling flow pattern. During this period, both inertial force and viscous force decrease gradually as a result of the velocity decrease at the melt front. However, the decreasing speed of the inertial force is larger than the viscous force because the reduced melt temperature increases the melt viscosity.

When the buckling flow arrives at the gate, the melt touches the upper and lower mold walls. Due to the no-slip boundary assumption the shear rate increases abruptly from 5.83 to 100 1/s near the mold walls. This leads the viscous force to increase to 64,523.6 Pa, far beyond the inertial force of 1568.3 Pa. Thus, the viscous force dominates the polymer melt progression and the melt filling switches to the conventional sequential filling mode.

### 4.2. Injection Speed

**Figure 3:**
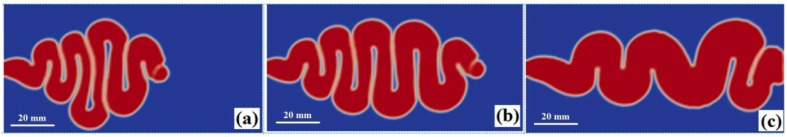
The buckling flow evolution varies with injection speeds of (**a**) 100 mm/s (*t* = 1.85 s); (**b**) 200 mm/s (*t* = 1.35 s); and (**c**) 300 mm/s (*t* = 0.95 s).

The influence of injection speed on jetting lengths and buckling frequency are shown in Figure 3. Jetting distance increases with the injection speed; high injection speed induces a long jetting distance. It increases from 61.5 mm (100 mm/s) to 120 mm (300 mm/s). When the injection speed exceeds a critical value (263 mm/s in this study), the melt pillar shoots directly to the opposite cavity, then the melt front folds and progresses as a buckling flow. On the other hand, Figure 3 shows that a higher injection speed does not increase the buckling swinging frequency but expands the buckling diameter instead. Since the high injection speed means more polymer volume is injected into the cavity at a time, this increases the viscous force and raises melt swinging resistance, consequently. Thus, the frequency and amplitude of the buckling for high injection speed is less than that at low speed.

**Figure 4:**
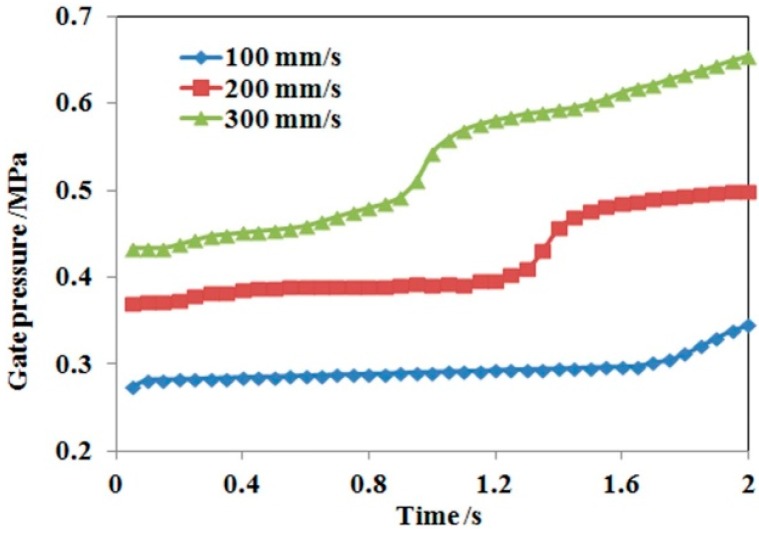
Gate pressure as a function of time at three injection speeds.

Figure 4 shows the variation of gate pressure with time under three injection speeds. During jetting and buckling stages, the melt only exchanges heat with cavity air. Since air thermal conductivity is far less than steel, the polymer melt does not lose much heat during this period. This leads the melt viscosity to not increase significantly, so the gate pressure increases slowly and the profile approximates a horizontal line for a low injection speed. When the melt touches the mold wall, the melt viscosity increases significantly due to the abrupt temperature decrease, leading to a sharp increase on the gate pressure profile for all injection speeds. Then the melt temperature changes smoothly and the gate pressure increases steadily and regularly. Figure 4 also indicates that the gate pressure increases with injection speed, which is in agreement with the normal filling rule.

### 4.3. Melt Temperature

**Figure 5:**
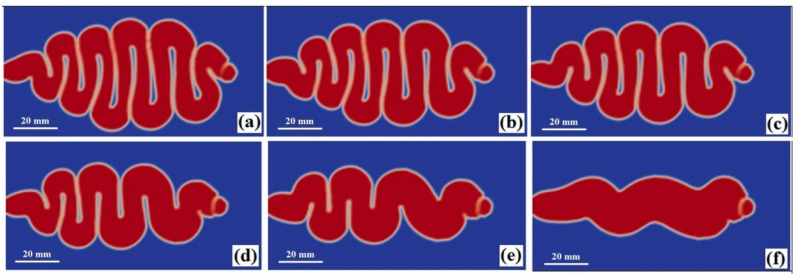
Buckling flow shapes at the end of jetting at melt temperatures (**a**) 235 °C (1.55 s); (**b**) 245 °C (1.45 s); (**c**) 255 °C (1.35 s); (**d**) 265 °C (1.2 s); (**e**) 275 °C (1.1 s); and (**f**) 285 °C (0.95 s).

The numerical simulations were conducted at melt temperatures 235, 245, 255, 265, 275, and 285 °C, and the other processing conditions are same as Section 4.1. The filling patterns for these melt temperatures are similar. The melt firstly jets into the cavity, then changes to a buckling flow, and finally switches to a conventional filling mode (see Figure 5). The simulated results shows that melt temperature does not have a significant effect on jetting distance. The difference between the longest jetting distance (Figure 5f) and the shortest distance (Figure 5a) is only 2.3 mm. However, the swinging frequency of the buckling decreases with melt temperature; for example, the frequency decreases from 10 for 235 °C to 4 for 285 °C. On the other hand, the average buckling diameter increases from 6.7 mm for 235 °C to 13.5 mm for 275 °C. Melt viscosity and the associated flow resistance decrease with temperature, so more melt is injected into the cavity for higher melt temperatures. This enlarges the buckling diameter and reduces the swinging frequency, as illustrated in Section 4.2.

### 4.4. Gate Location

**Figure 6:**
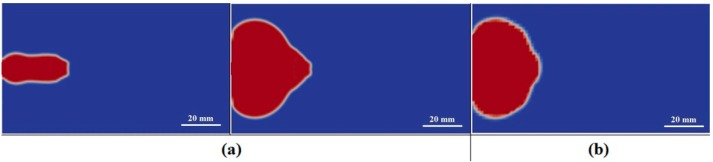
Melt shapes near the gate in the initial filling stage for gate locations at (**a**) 1 mm; and (**b**) 0 mm away to mold wall.

The gate location effect on jetting was investigated by moving the gate 1 mm and 2 mm (contact with the boundary) to the lower mold wall, respectively. The simulations were performed with the same process conditions as Section 4.1. The simulated melt filling for these two gate locations are shown in Figure 6. When the gate approaches the mold wall (1 mm gap), jetting still occurs but the jetting length reduces significantly compared with that of middle gate location. Then the melt flow switches to the sequential filling at once without a buckling flow. Since jetting is unstable due to gravity, inertial force, and air resistance, the melt touches the mold wall easily within the shortened gap between the gate and mold wall. Once the melt contacts with the mold wall the viscous force increases abruptly, as illustrated above. The enlarged viscous force makes the melt filling in sequential mode. When the gate is located at the mold wall, the viscous force predominates from the beginning of melt filling, prohibiting the jetting as the filling commences.

### 4.5. Experimental Verification

The precision injection molding machine (Demag 80 t, Terex Corporation, Wetter, Germany) was used to manufacture the studied plate product. The melt and mold wall temperatures were set at 255 and 55 °C, respectively, the injection pressure is 500 bar, filling/packing switches at 95% filled volume, and injection velocity of the screw is 40 mm/s, which is approximately 200 mm/s of melt velocity at the gate, according to the equal volume equivalence.

**Figure 7:**
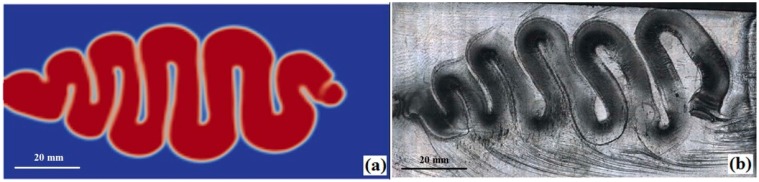
Comparison between simulated jetting (**a**) and experimental jetting (**b**) for the plate part molding.

Figure 7 shows the simulated buckling at the end of jetting and the photograph of short shoot injection experiment under the same process conditions. This figure indicates the simulated buckling is in good agreement with the experimental result. Both of the swinging frequencies are 10, and the difference between the two jetting distances is only 1.5 mm (1.41% of the experimental jetting distance). Moreover, the average relative difference of the swinging amplitudes of the middle and the rear (near gate) parts is 4.53%. However, this value exceeds 8.2% for the front buckling. This is due to the inaccurate prediction of the melt front temperature. In this study, the heat transfer coefficient between the melt and the air is set to 1250 W/(m^2^∙K) (Moldflow suggested) which may be larger than the actual value. Thus, the simulated the melt front temperature may be lower than the real melt temperature, which increases the viscous resistance for buckling swinging. Since the times of the melt contacting the air in the middle and rear jet are shorter than that of the front jet, the decreased temperatures in these two sections are less than that of the front and the simulated difference does not induce significant influence on the buckling flow.

## 5. Conclusions

Conventional injection molding simulation cannot simulate jetting, buckling, and other special phenomenon for ignoring the influence of the inertial force. In this study, a full three-dimensional melt flow model was established based on an incompressible, viscous, and non-isothermal fluid. The finite volume method was employed to discretize the mass, momentum, and energy equations. A program was developed to simulate the influences of injection processes and mold structure on jetting. For viscous fluid it can be concluded as following:

1. If the inertia force competes over the viscous force at the melt front, jetting occurs. When viscosity exceeds the inertial force, the jetting switches to buckling, and finally changes to traditional sequential filling.

2. Gate location is the key factor for jetting, when the gate faces to the cavity directly and is located enough of a distance away from the mold wall, jetting can occur.

3. Injection speed has a significant effect on jetting and buckling, as high injection speed leads to a longer jetting length and a lower swinging frequency.

4. In the processing temperature scope, melt temperature has little effect on the jetting length, but a high melt temperature decreases the swinging frequency and increases the buckling diameter.

## Figures and Tables

**Table 1:** Material parameters of polypropylene, Polyfort FIPP MKF 4025.

Seven-constant viscosity	Other
n=0.3263	$\rho =1024.7\;\text{kg}/m^{3}$
$\tau^{*}=19274.6\text{ Pa}$	$C_{p}=2143\;J/\left(\text{kg}\cdot ℃\right)$
$A_{1}=33.685$	k=0.25 W/(m⋅℃)
${\tilde{A}}_{2}=51.6\;K$	
$D_{1}=1.01748e+15\text{ Pa}\cdot s$	
$D_{2}=263.15\;K$	
$D_{3}=0\;K/\text{Pa}$	
